# What we say and what we do: The relationship between real and hypothetical moral choices

**DOI:** 10.1016/j.cognition.2012.02.001

**Published:** 2012-06

**Authors:** Oriel FeldmanHall, Dean Mobbs, Davy Evans, Lucy Hiscox, Lauren Navrady, Tim Dalgleish

**Affiliations:** a Cambridge University, Cambridge CB2 1TP, UK; b Medical Research Council Cognition and Brain Sciences Unit, Cambridge CB2 7EF, UK

**Keywords:** Morality, Real moral decision-making, Contextual information, Motivational factors

## Abstract

Moral ideals are strongly ingrained within society and individuals alike, but actual moral choices are profoundly influenced by tangible rewards and consequences. Across two studies we show that real moral decisions can dramatically contradict moral choices made in hypothetical scenarios (Study 1). However, by systematically enhancing the contextual information available to subjects when addressing a hypothetical moral problem—thereby reducing the opportunity for mental simulation—we were able to incrementally bring subjects’ responses in line with their moral behaviour in real situations (Study 2). These results imply that previous work relying mainly on decontextualized hypothetical scenarios may not accurately reflect moral decisions in everyday life. The findings also shed light on contextual factors that can alter how moral decisions are made, such as the salience of a personal gain.

## Highlights

► We show people are unable to appropriately judge outcomes of moral behaviour. ► Moral beliefs have weaker impact when there is a presence of significant self-gain. ► People make highly self-serving choices in real moral situations. ► Real moral choices contradict responses to simple hypothetical moral probes. ► Enhancing context can cause hypothetical decisions to mirror real moral decisions.

## 1 Introduction

Bertrand Russell captured the nature of human morality when he stated “we have two kinds of morality side by side: one which we preach but do not practice and another which we practice but seldom preach” (Russell, 2004). Despite this capacity for moral hypocrisy, adhering to a human moral code—a set of complex, evolved, and learned capacities (Wright, 1994)—is integral to promoting pro-social interactions. These interactions are guided by deeply ingrained moral proscriptions, perhaps the most important of which is not to harm others (Cushman, Young, & Hauser, 2006; Haidt, 2007). Aversion to harming others is a moral imperative that not only transcends cultures (Brown, 1991), but also species (Masserman, Wechkin, & Terris, 1964). It is so strongly held that it can drive our hypothetical moral choices, even when a readiness to inflict harm would serve the greater good (Foot, 1978). In fact, aversion to harm appears to intensify as the prospect of hurting another becomes increasingly ‘up close and personal’ (Greene, Sommerville, Nystrom, Darley, & Cohen, 2001), with people more likely to avoid inflicting harm if they have to carry out the physical act themselves. This is illustrated most starkly by people’s responses to moral scenarios like the classic Trolley dilemmas. In these hypothetical moral problems, participants are willing to acquiesce to killing another person in order to save the lives of five others if it requires flipping a switch to change the direction of the out-of-control trolley (Trolley Dilemma). However, in the Footbridge Dilemma, there is an almost universal refusal to contemplate killing another by physically pushing them in front of the trolley even if the act would save five others (Foot, 1978; Greene et al., 2001).

But do people practice what they preach when it comes to averting harm in real life? While this moral proscription to not harm others has been repeatedly shown to underpin hypothetical moral decisions (Cushman et al., 2006; Greene, Nystrom, Engell, Darley, & Cohen, 2004; Waldmann & Dieterich, 2007), it is unclear how much it motivates real moral choices, especially when those choices directly involve the option for self-benefit. In fact, daily life and human history are rife with examples of people being harmed in exchange for personal gain or higher ideals. So what can explain this apparent disconnect between the data from decades of research on human morality (Greene & Haidt, 2002; Haidt, 2001; Kohlberg, 1973; Moll, Zahn, de Oliveira-Souza, Krueger, & Grafman, 2005) and anecdotal evidence amassed over hundreds of years of historical record?

A key factor is that research on human morality has largely focused on hypothetical moral reasoning. Most commonly, subjects are presented with hypothetical moral vignettes (Cikara, Farnsworth, Harris, & Fiske, 2010; Crockett, Clark, Hauser, & Robbins, 2010; Greene et al., 2001; Greene et al., 2004; Kahane et al., 2011; Tassy et al., 2011), stripped of all non-essential contextual information, and then queried on their intention to perform an action. These stylised scenarios act as a barometer for subject’s moral knowledge and the responses can be taken as a rough indication of subjects’ readiness to perform the associated moral behaviour (Ajzen, Brown, & Carvajal, 2004).

The reliance on these kinds of hypothetical moral probes potentially presents limitations to understanding moral cognition. First, philosophical dilemmas such as the Trolley and Footbridge scenarios capture a particular kind of moral tension, where the choice to harm another is offset against the ‘greater good’ (i.e. saving five lives at the cost of one). It is arguably rare in the real world to be faced with the kinds of utilitarian decisions encapsulated in these classic dilemmas (Knutson et al., 2010), where the welfare of many is pitted against one’s reluctance to commit a personal act of violence. We contend that most moral choices that face us in daily life pit the fundamental motivation of not harming others (physically or psychologically) against that of maximising self-gain (Haidt, 2007), and that this distinct class of moral tension is especially prevalent in everyday life. Accordingly, the motivational force of self-benefit is poorly understood, as research to date has largely steered clear of using probes encapsulating this prototypical moral conflict.

Second, using hypothetical probes assumes that there is a strong link between the moral intentions that they elicit and action in the real world (Teper, Inzlicht, & Page-Gould, 2011). But the stories of lying, cheating, and stealing that fill newspapers and history books suggest little congruence with the psychological data demonstrating people’s reported aversion to harming others. So why might the link between people’s stated moral intentions and their action be weaker than often presumed? Moral cognition, like any dynamic system, relies on complex cognitive processes that integrate socio-emotional factors, such as situational cues, past experience, and potential future rewards or consequences (Bandura, 1989). Indeed, research on the psychology of choice has illustrated that decisions are influenced by reward and punishment contingencies (Tversky & Kahneman, 1981), as well as the environment in which they are made (Isen & Levin, 1972). To ascertain if hypothetical moral probes act as a good proxy for real moral behaviour, moral cognition needs to be studied in action-relevant environments where the stakes are immediate (Kang, Rangel, Camus, & Camerer, 2011), emotionally charged (Teper et al., 2011), and tangible.

The central aims of the studies reported here were to shift the focus of psychological inquiry by investigating moral decision-making in situations where harm to another and significant personal gain act in direct opposition. And then to examine how this moral tension was resolved in both hypothetical and real contexts.

## 2 Study 1

Since the literature reports that aversion to harm is such an influential force in hypothetical moral decisions (Cushman et al., 2006), our first assumption was that this deeply seated moral imperative would dictate peoples’ predictions of human moral choices. To test this, in Study 1a we asked people whether they thought future participants would be more or less likely to avoid harming another for significant personal gain if the stakes were real compared to hypothetical. Our prediction was that subjects would postulate that in real moral scenarios people would be more likely to abide by the harm principle than those imagining doing the same scenario. Study 1b then examined whether the views of the survey sample were borne out: we directly compared real and hypothetical decisions in two experimental conditions. Our prediction was that when motivational forces are concrete and real rather than presented in a hypothetical scenario, the incentive for significant self-benefit would become more compelling than the proscription not to harm others. In short, while people in our survey (Study 1a) might predict that the aversion to harming others would be more influential in real moral situations compared to hypothetical ones, an experimental test (Study 1b) would reveal the opposite (Batson, Kobrynowicz, Dinnerstein, Kampf, & Wilson, 1997; Tetlock, 2003).^1^

**Fig. 1:**
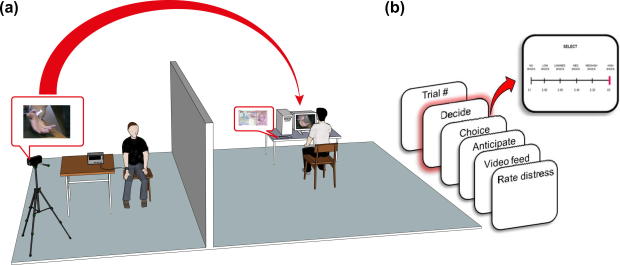
(a) Experimental setup of the Real Pain versus Gain task. The Decider and Receiver (a confederate) met and interacted before the start of the experiment, during which the Decider was also given a low-level sample shock. The video feedback was actually pre-recorded videos of real shocks being administered. (b) Trial sequence for the Pain versus Gain task with highlighted decision event.

To test our hypotheses, we used both real and hypothetical versions of a ‘Pain versus Gain’ paradigm (PvG; Fig. 1a) where we asked participants (Deciders) to make a moral choice: benefit oneself financially or prevent physical harm to another (the Receiver). Deciders chose how much, if any, of a £20 endowment they would spend to prevent a series of painful electric shocks from reaching the Receiver. The more money Deciders paid on a given trial (up to £1 per trial—20 trials), the lower the shock level inflicted. Paying £1 removed the shock altogether; paying nothing ensured the highest shock level. The outcome variable was how much money Deciders kept across 20 trials (Money Kept), with lower amounts indicating that preventing harm was prioritized. Deciders watched the shocks being inflicted via video feed (Fig. 1b). Importantly, Deciders were also informed that all money remaining after 20 trials would be randomly multiplied to a potential maximum pay-out of £200. This was designed to make the potential personal gains on offer significant and motivationally compelling.

### 2.1 Study 1a: A survey predicting moral behaviour

#### 2.1.1 Methods

##### 2.1.1.1 Participants

We recruited 88 participants (mean age 30.8, and SD ± 0.6; 35 females) for the survey. For this study and for the others reported here, participants were recruited from the volunteer panel at the Cognition and Brain Sciences Unit, Cambridge UK, and from the postgraduate student community in Cambridge. Participants in the studies were compensated for their time and travel and allowed to keep any earnings accumulated during the task. Participants gave informed consent, and the study was approved by the University of Cambridge, Dept. of Psychology Research Ethics Committee.

##### 2.1.1.2 Materials and procedure

Participants were asked to complete a survey questionnaire regarding their predictions of what other participants would do during both Real and Hypothetical versions of the PvG task described above. The PvG task was outlined and the survey participants were explicitly asked whether other (future) subjects participating in our studies would inflict more or less harm on the PvG task when carrying it out for real as compared to simply imagining carrying it out.

#### 2.1.2 Results and discussion

As expected (Gilbert & Wilson, 2007; Wilson, Wheatley, Meyers, Gilbert, & Axsom, 2000), the significant majority of subjects surveyed (74%) thought that the proscription to not harm others would exert a greater influence during the real condition, resulting in people keeping less money in the Real PvG compared to the Hypothetical PvG.

### 2.2 Study 1b: An experimental test of moral behaviour

The next question was whether these predictions of human moral behaviour (identified in our survey; Study 1a) were borne out in real moral action. To look at this, in Study 1b we asked one group of subjects to make a hypothetical judgment about how much money they would imagine keeping in the PvG task. We then compared their judgments to the responses of those completing a Real version of the PvG task. Our prediction was that, counter to the consensus view held by our survey sample, those participants completing the Real PvG would prioritize personal gain over another’s physical welfare to a greater extent than participants engaging with the Hypothetical PvG.

#### 2.2.1 Methods

##### 2.2.1.1 Participants

Forty-six participants completed Study 1b; 20 (mean age 26.6, and SD ± 4.3; 9 females) completed the Real PvG task and 26 (mean age 25.5, and SD ± 2.0; 18 females) completed the Hypothetical PvG scenario. Four additional participants were excluded from analyses due to expressing significant doubts about the veracity of the Real PvG task on a questionnaire administered at the end of the experimental session (see below).

##### 2.2.1.2 Materials

###### 2.2.1.2.1 The Real PvG task setup

In order to avoid priming moral attitudes and to minimise explicit moral reasoning during task performance, we recruited subjects under the pretence of participating in a psychology study investigating economic decisions. We also went to great lengths to ensure that our participants believed the paradigm, including the use of detailed experimenter scripts and meticulous and comprehensive experimental set up. Prior to the task, each subject and our confederate (who took the role of Receiver) completed paperwork where subjects provided consent both to receive and administer electric shocks. Both participants (true subject and confederate) were told that they were recruited from two different panels—one subject preselected to be the Decider (the true subject administering the shocks) and the other to be the Receiver (the confederate receiving the shocks). In order to introduce a personal element to the task context and to enhance the believability of the protocol, the Decider and Receiver were together and allowed to interact. They were then both taken to the testing laboratory that housed the electric shock generator, a Digitimer DS7A, and briefed on the set up of the experiment. The Decider, sitting in the place where the Receiver would subsequently sit for the duration of the experiment, received the low-level shock choice and was asked to rate his/her own pain on a 10-point scale (anchored from 1 = no pain to 10 = extreme pain). This was to provide the Decider with explicit information concerning what the Receiver would later experience during the Real PvG task. The Decider was then taken to another room while the Receiver was purportedly connected to the shock generator (see Fig. 1a). Once there, the Decider was endowed with a real £20 note and told that the money could be used to stop or attenuate the shocks planned for the Receiver. Effectively, the more money the subject paid, the lower the shock level the other participant received on a given trial. Consequently to stop all of the shocks across all 20 trials, Deciders would need to spend all £20.

During the PvG task subjects believed they were viewing in real time actual shocks being administered to the Receiver, who was sitting in a nearby testing laboratory, via a video feed. However, the videos were in fact pre-recorded films of actual shocks being administered to the confederate, pre-rated by an independent group so as to be matched for shock level and corresponding pain intensity.

###### 2.2.1.2.2 Stimuli

The Real PvG task comprised a series of 8 screens per trial across 20 trials (see Fig. 1b). Each trial began with a screen displaying the running amount of the subject’s bank total (£20 on Trial 1) and current trial number. Subjects then had to decide upon, and use a visual analogue scale (VAS) to select the amount of money they wanted to spend on that trial and thus the corresponding shock to be administered to the Receiver. This phase was partitioned into the “decide” and “select” periods. The Decide screen was presented for a fixed 3 s during which subjects were asked to think about their decision. The select screen was self-paced. After making a selection, subjects saw a 3 s display of their choice before experiencing a 4 s anticipation phase during which subjects were told their choice was being transmitted over the internal network to the adjacent testing lab where the Receiver was connected to the shock generator. Following this anticipation period, subjects viewed a 4 s video of the shock being administered to the Receiver, or no shock if they had opted to spend the full £1 permitted on a given trial. During this video feed, subjects viewed the Receiver’s hand reacting to the choice shock. Finally, subjects used a seven point VAS to rate their distress levels on viewing the consequences of their decision, before viewing a 4 s inter-trial-interval (ITI). At the conclusion of the 20 trials, subjects were able to press a button to randomly multiply any remaining money between 1 and 10 times.

###### 2.2.1.2.3 Shock administration

A Digitimer DS7A, which is a Food and Drug Administration (FDA) approved device for both experimental and clinical settings, was used to administer the shocks in the Real PvG. Subjects received a mild, non-harmful sample electric shock via an electrode dipped in saline solution placed on the underside of their right wrist. While subjective pain level is different for everyone, a barely perceptible, non-painful shock would approximately be 50 μs with 2.5 mA at 400 V; subjects received a ‘low’ level sample shock at 2.5 mA with 200 ms at 400 V prior to their role as Decider.

###### 2.2.1.2.4 Post-experiment questionnaire

After the experimental session was finished for the Real PvG condition, subjects answered a series of questions that asked them to indicate on 8 point Likert scales: (1) whether they felt they were being watched during the experiment (anchored 1 = always watched, 8 = never watched). This was in order to examine any putative effects of reputation management on responses; (2) how much responsibility they (as Deciders) had for the electric stimulations administered (anchored 1 = no responsibility, 8 = full responsibility). This was to verify that participants did feel responsible for their choices, and also to explore whether degree of reported responsibility was associated with the behavioural outcomes; (3) and whether subjects had any doubt as to the veracity of the paradigm (anchored 1 = completely believed, 8 = did not believe). This was to allow us to exclude participants who indicated disbelief in the protocol (those scoring above the mid-way point on the scale; see below). We also checked if subjects were aware of Milgram’s famous Obedience study (Milgram, 1963) as we wanted to able to explore whether such knowledge had an influence on participants behaviour.

###### 2.2.1.2.5 Hypothetical Scenario PvG task

Subjects in this condition completed a contextually impoverished written version of the PvG probe (Scenario PvG). The aim was to use the same format favoured by the majority of the literature on moral reasoning (Greene et al., 2001). Subjects were asked to imagine a scenario in which they and another volunteer have both agreed to participate in a psychology experiment. They are told that in this scenario they have been given £20 at the beginning of the experiment and informed that any money they have left at the end can be multiplied up to 10 times with a possible pay out of £200. This is money they can keep. Participants are then told that their task is to make decisions regarding the number and intensity of painful but harmless electric shocks the other volunteer will receive. They are informed that they can spend their £20 to reduce the pain of the shocks or to stop the shocks from reaching the other volunteer altogether. Having imagined this situation, participants are asked whether they will spend their £20 to ensure no shocks reach the other volunteer, or whether they will keep the money at the expense of the other participants’ physical welfare.

#### 2.2.2 Results and discussion

Participants in the Real PvG and Scenario PvG groups were matched for age (*t*(44) = 0.46, *p* = 0.65), however, the groups were not matched for gender ratio: consequently our key analysis was repeated with gender as a covariate.

**Fig. 2:**
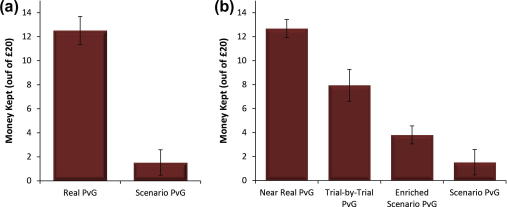
(a) Money retained across Study 1b shows that subjects, when responding to a written hypothetical version of the PvG task highly prioritise the Receiver’s physical welfare as compared to those in the Real condition who maximise their own self-interest. (b) Money retained across Study 2 illustrates that as more contextual information—like the specific nature of the reward and harm contingencies of the decision—became available to subjects, hypothetical moral probes better predicted real moral decisions.

In Study 1a, our survey sample strongly endorsed the view that future participants in the PvG task would be more likely to preserve the welfare of the Receiver (and thus keep less money for themselves) in the real condition than in the hypothetical condition. However, the results of Study 1b (Fig. 2a) were in stark contrast to this. The data showed that Deciders kept most of their endowment in the Real PvG condition (mean: £12.52/£20; SD ± 4.8) and more than seven times as much as the participants completing the hypothetical Scenario PvG task (mean: £1.53/£20; SD ± 5.43; [*t*(44) = 7.12, *p* < 0.0001, Cohen’s *d* = 2.05]), where nearly all of the endowment was relinquished to protect the Receiver from harm. This difference remained significant when gender was included as a covariate (*F*(1, 43) = 37.3, *p* < 0.0001). In fact, only 7% of the subjects in the Scenario PvG condition said they would keep any portion of the money at the Receiver’s expense. In contrast, in the Real PvG condition 100% of subjects kept a portion of their original £20.

There was no support for Money Kept being significantly associated with degree of belief in the veracity of the paradigm (*r*(18) = 0.28, *p* = 0.24; mean believability score on a 8-point likert scale: 1.85, SD = ±0.93). However, since the believability of the PvG paradigm is paramount in interpreting the results, we also analysed the data from subjects (*n* = 15) who rated the PvG paradigm as only a 1 or 2 on the 8-point believability scale. These subjects kept a comparable amount of money as the full sample of 20 (mean = £11.75, SD = ±4.8), and significantly more than the hypothetical scenario group (*t*(39) = 6.01, *p* < 0.0001, Cohen’s *d* = 2.04). There was still no significant correlation between beliefs and Money Kept for this group (*r*(13) = .23, *p* = 0.41).

Importantly, there was no evidence that subjects modified their moral decisions on the Real PvG task in response to reputation management—as indexed by feelings of being watched (Landsberger, 1958)—since the relevant post study rating did not correlate significantly with Money Kept (*r*(13) = −0.32; *p* = 0.45: responses only acquired for 15 of the 20 subjects). Additionally, for the Real PvG, participants rated themselves more responsible than either the Experimenter or the Receiver for the pain inflicted on the Receiver. There were no significant differences in correlations between Money Kept and degree of responsibility (*r*(18) = 0.20, *p* = 0.41; mean responsibility ratings on a 8-point likert scale: 6.35, SD ± 1.53). Furthermore, awareness of Milgram’s Obedience study did not correlate significantly with Money Kept (*r*(18) = −0.33, *p* = 0.15). In fact, only six subjects reported being aware of Milgram’s study and greater awareness was in the direction of being associated with keeping less money. One might suppose that knowledge of the Milgram experiment might reduce participants’ belief in the current study, however, the data illustrates the opposite association. Finally, Deciders’ subjective pain ratings of the sample shock revealed that they found the lowest shock uncomfortable, and these ratings did not correlate significantly with Money Kept (*r*(18) = −0.22, *p* = 0.35; mean pain ratings on a 10-point likert scale: 3.25, SD ± 1.97).

The findings from Study 1 illustrate that the proscription to not harm others—predicted by our survey sample to be a powerful force in real life moral decisions—in fact has surprisingly little influence when potential significant personal gain is at stake. It seems that when a decision is entirely hypothetical, the influence of breaking a moral imperative (it is morally wrong to harm others, especially for monetary gain) is more compelling than the influence of imaginary monetary benefit. In comparison, when this substantial monetary self-benefit becomes tangible and real, it appears that the aversion to harming others is no longer the most salient feature within the decision-making space. Imaginary moral scenarios, stripped of contextual and motivational cues like concrete incentives and consequences, seem to maximise people’s opportunity to adhere to moral duties, such as not harming others (Tversky & Kahneman, 1974; Tversky & Kahneman, 1981). In contrast, when the moral choice is made real, it appears that the influence of actual ‘money on the table’ is the overriding influence. These data demonstrate that there can be fundamental differences between how we assume people will act (as indexed by the survey data) and how they actually act, and that introducing real outcome contingencies can drastically change moral behaviour.

One potential limitation of the experimental framework used in Study 1b is that subjects in the Real PvG condition were recruited under the pretext of completing a psychology economic task, while subjects in the hypothetical Scenario PvG condition were told to imagine partaking in a psychology experiment where they must make decisions about shocks and money. However, the effect size of the differences between conditions was sufficiently large that, while this issue must be taken seriously, we feel that it cannot fully account for the data.

Another issue that merits careful consideration is the believability of the Real PvG paradigm. It is important that participants were not keeping more money in the real condition simply because they did not believe that real shocks were being administered to the Receiver, and therefore felt that there was no disincentive to keep the money. There are a number of reasons why we feel that this is unlikely to be the case. First, we were careful to probe participants about their belief in the paradigm. We felt confident that they would respond to this question honestly because if they had behaved in a self-serving manner due to the assumption that the shocks were not real, we reasoned that they would want to inform us of this to avoid being judged as ‘immoral’.^2^ Furthermore, we excluded from the analyses participants who expressed any disbelief in the protocol. Indeed, in supplementary analyses (see above) we focused only on participants who scored very lowly on the belief scale, and found comparable results to the analyses with the initial sample. Additionally, there was no support for a significant association between belief ratings and Money Kept.

Second, we would also reiterate that we went to great lengths to enhance the believability of the task using carefully scripted experimental procedures, ensuring that the participants met and interacted with the Receiver, and viewed the room where the shocks would be delivered. We also delivered a sample shock to each subject. Finally, although pre-recorded, the video feedback participants saw was footage of actual shocks being delivered.^3^

Third, we sought to address the concern that prior knowledge of Milgram’s obedience studies, which used bogus shock administration, might compromise the present study. We therefore took care to ask participants about their knowledge of the Milgram study and to examine whether this influenced the results. Knowledge of the Milgram experiment (six subjects) did not appear to have been a factor in their responses. For example, one subject said: “*Yea I know about Milgram. And I thought about that when you were describing the experiment, but I thought you couldn’t do that anymore so I knew these shocks were real. Plus I felt it [the shock] and I saw myself being recorded when I was in the other room.*” In sum, we feel confident that for the participants in our analysis sample from the Real PvG condition, disbelief in the paradigm was not a significant issue.

One possible account of the findings from Study 1 is that simple hypothetical moral scenarios maximise the need for participants to mentally simulate the pros and cons for a given decision. In such situations, the impact of ingrained moral proscriptions (even when there is knowledge that the decision is hypothetical) is arguably far stronger than the impact of imaginary money. In contrast, in the real situation, the need for mental simulation is minimised, allowing the actual motive power of real money and real harm to yield their influence such that if the money is sufficient, the harm will be administered. This analysis is consistent with Loewenstein’s work on the hot–cold empathy gap (Van Boven & Loewenstein, 2005) and with Damasio’s somatic marker hypothesis (Dunn, Dalgleish, & Lawrence, 2006), both of which emphasise the differential influence of emotion-arousing features of a decision space in real decisions compared to hypothetical ones.

It follows from these arguments that reducing the need for mental simulation on the PvG task, even in situations where the money and harm remain hypothetical, should shift participants’ behaviour away from that exhibited in the simple scenario condition of Study 1b and closer to that exhibited in the Real PvG task (Wilson et al., 2000).

## 3 STUDY 2: Enriching the context

Based on this reasoning, in Study 2 we created three additional hypothetical PvG tasks, with stepped increases in the richness of their situational and contextual cues: a longer written version of the hypothetical task used in Study 1b (Enriched Scenario PvG), a computer-administered version where participants actually worked through the 20 trials knowing that the task was hypothetical but did not meet the Receiver or experience a sample shock (Trial-by-Trial PvG), and a ‘near-real’ version that was identical to the Real PvG in every respect except that the money and shocks were completely imaginary (Near-Real PvG). Our hypothesis was that there would be a significant increase in Money Kept across the 4 versions of the hypothetical PvG (the simple scenario from Study 1b and the three new versions) as the versions reduced the scope and need for mental simulation when making a moral decision.

### 3.1 Methods

#### 3.1.1 Participants

In Study 2, 93 subjects completed the three new hypothetical versions of the PvG task: 15 subjects (mean age 25.8, and SD ± 1.1; 7 females) completed the Near-Real PvG, 18 subjects (mean age 23.8, and SD ± 1.0; 12 females) completed the Trial-by-Trial PvG, and 63 subjects (mean age 29.0, and SD ± 2.2; 41 females) completed the Enriched Scenario PvG.

#### 3.1.2 Hypothetical PvG conditions

##### 3.1.2.1 The Near-Real PvG

This condition required subjects to complete the same task as in the Real PvG (see Study 1b). Deciders still met the Receiver and experienced the same low-level shock. The only differences were that Deciders were not endowed with money and were told to simply imagine making the relevant decision. Thus, in this condition there was no video feedback but instead a blue screen was presented for 4 s each trial.

##### 3.1.2.2 Trial-by-Trial PVG

This version was the same as the Near Real PvG except that subjects did not have the experience of meeting the Receiver or receiving a sample shock. Again, subjects were asked to imagine that £20 was at stake and that another participant was receiving the chosen shocks. A blue screen was also presented for 4 s during the video feedback portion of each trial.

##### 3.1.2.3 Enriched Scenario PvG

Subjects were presented with a contextually rich written version of the PvG dilemma in which more information was provided than in the simple scenario in Study 1a.

### 3.2 Results and discussion

The data from the three new hypothetical scenarios are combined with the data from the simple scenario in Study 1b. The groups of participants across the four conditions were matched on age and gender ratio (age: *F*(3,118) = 0.92, *p* = 0.43; gender: Chi-square = 2.9, *p* = 0.39).

The data from the four hypothetical PvG conditions are shown in Fig 2b. ANOVA revealed a clear and significant effect of context, such that systematically enriching the situational context, and thus increasing the ecological validity of the dilemma, reduced the gap between hypothetical moral choices and real moral action [(see Fig 2a), *F*(3,118) = 16.24, *p* < 0.0001, partial *η*^2^ = 0.29; with each condition significantly differing from its neighbour: post hoc LSD tests Ps < 0.05]. A comparison between the Near-Real PvG condition in Study 2 and the Real PvG in Study 1b revealed no significant difference between them [*t*(33) = −0.11, *p* = 0.91]. This supports our contention that the more a hypothetical PvG task contextually resembles the Real PvG, the more people are willing to augment their personal gain at the expense of harming another.

## 4 General discussion

Classical research on morality has shown that the proscription not to harm others is a deeply ingrained moral principal (Greene et al., 2001). This was reflected in participants’ predictions about moral decisions to inflict harm in exchange for significant monetary gain: Study 1a showed overwhelmingly that participants believed people would be much less likely to inflict harm for money when the consequences were real than when they were simply hypothetical. However, a test examining these predictions (Study 1b) revealed the opposite result: in contrast to the survey predictions, participants were significantly more likely to inflict harm for monetary gain under real conditions than they were under hypothetical conditions. We reasoned that the influence of harm aversion diminishes as the impact of other motivational forces—such as significant financial gain—become more salient. These findings imply that not only are subjects unable to accurately judge outcomes of moral behaviour, but under certain conditions they also make starkly different moral choices in real, compared to hypothetical, contexts.

To explain why real moral choices radically contradict the responses generated by the type of decontextualized hypothetical moral scenarios used in Study 1b, we explored whether enriching the contextual information available to subjects would influence their decisions (Study 2). We found that the more spartan the contextual information, and hence the more room people have for mental simulation when addressing a hypothetical moral problem, the more subjects’ responses diverged from real behaviour. This strongly suggests that the underspecified and impoverished nature of hypothetical moral probes is unable to capture the complex social, emotional and motivational pressures inherent to real moral decisions. This is consistent with work in other psychological domains (Gilbert & Wilson, 2007; Wilson et al., 2000), showing that simulating future choices can be markedly inconsistent with reality.

Accordingly, we found that enhancing the specificity and ecological validity of situational cues in hypothetical moral probes—thereby encapsulating the complex motivational forces at work in real decisions—can bring hypothetical choices in-line with real moral behaviour. Therefore, we suggest that during simple hypothetical moral decisions, subjects are unable to access the contextual-dependent knowledge experienced during real moral choices, and instead rely on their most salient motivation: to uphold the moral duty to avoid harming another.

Taken together, these findings have a number of important implications. First, and most strikingly, peoples’ real moral choices drastically contradict the responses generated by simple hypothetical moral probes. Second, these data suggest that our moral beliefs may have a much weaker impact on our decision-making if the context is enriched with other compelling motivational forces, such as the presence of a significant self-gain. This raises questions about whether hypothetical moral decisions generated in response to decontextualized scenarios, act as a good proxy for real moral choices. Even though moral ideals are strongly ingrained within society, actual moral choices seem exceptionally labile and profoundly influenced by the tangibility of rewards, situational cues and concrete consequences.

While the aim of the studies reported here was to examine considerations of harm against self-benefit under both real and hypothetical conditions, many questions arise from this data: what are the cognitive mechanisms driving self-serving behaviour? How do individual differences contribute to the moral decision-making process? What other motivations besides harm aversion influence our sensitivity to self-benefit? Future studies designed to disentangle the various cognitive mechanisms driving moral motivations is an important next step in further elucidating the nature of moral behaviour.

## Footnotes

1 An important aspect of these predictions is that we are referring to situations where harm aversion is pitted against *significant* personal gain. For the majority of people, making any choice to harm another has a personal cost, as it comes with an awareness that a moral code has been violated (not to mention the potential social distress associated with being caught making such a choice). We contend that for personal gain to drive such a choice, it has to be substantial enough to offset potential moral pain and social opprobrium (cf. Teper et al., 2011).

2 In fact, subjects’ comments to us after the task, but before debriefing, indicate that subjects felt a genuine motivational conflict. For example, one subjected stated: “I struggled with what to do. I wanted the money but I didn’t want to hurt him. I decided that he could take a little pain and I could make a little money. Somewhere in the middle. But I felt guilty”. Another said: “I was curious to see how ruthless I could be. I was happy to see that I could control myself. I wanted the money. I tried the highest shock first. I wanted to see the reaction. And then took it from there.”

3 In addition, the nature of feedback during debriefing suggested that subjects—after being told the true nature of the study—truly believed the paradigm: “I am really upset that it is not real. If I had known it was real I would have kept all the money” and “I can’t believe it is not real. I found the shock painful. So I know that he must have been in real pain.”
